# Spirit of the British and American Periodicals

**Published:** 1840

**Authors:** 


### Spirit of the British and American Periodicals, &c.

### Some Pathological Facts.

In our Dublin contemporary* we have a full report of the Proceedings of the Pathological Society of Dublin—a very excellent society, whose meetings are attended by distinguished men, and enriched by valuable facts. Of the latter we shall cull the more important.

1. *Precautions in Operating for Empyema.*—Speaking of a case of empyema, Dr. Corrigan observed that the phenomena which attend the formation of matter externally are very apt to prove deceptive. The opening by which the fluid in the cavity of the pleura escapes in the first instance, is often very small, so small indeed, that it will barely admit the introduction of a probe, while the matter in the cellular tissue beneath the skin is often in very considerable quantity. The fluid which escapes from the pleural sac creates and keeps up irritation, and hence the quantity of pus in the external abscess is sometimes much greater than one would expect. When an incision is made, a large quantity of matter flows out, and the operator thinks he has made a sufficiently large opening. The lapse of four-and-twenty hours is sufficient to convince him of his mistake; the matter now either trickles out very slowly, or perhaps stops altogether, and continues so until a fresh opening is made. Dr. Corrigan said he mentioned this as a practical fact. In all cases of this kind, it is necessary to open, not only the external abscess, but also to take care, that the matter of the empyema has a ready mode of exit.

2. *Traumatic Emphysema without Laceration of the Pleura, or Fracture of the Ribs.*—A strong man was run over by the mail. On admission into the Richmond Hospital he had intense dyspnœa, with the usual symptoms which accompany internal hæmorrhage; there was also slight cough, but he did not spit any blood. All the muscles of the chest, abdomen, and neck were thrown into the most violent action, and the dyspnœa under which he laboured was frightful. His pulse was about 96, weak, and failing; his face pale and livid; his extremities cold. At the root of the neck there was a large tumor, which was found to be produced by effusion of air into the cellular substance, constituting subcutaneous emphysema; the whole of the anterior, lateral, and posterior parts of the chest became subsequently inflated. He died about three-quarters of an hour after admission. On dissection, the right lung was found as compressed as in an old emphysema, by an enormous quantity of air in the cavity of the pleura. There was a large quantity of extravasated blood lying about the roots of the great vessels and primary branches of the aorta. There were three different lacerations in the substance of the lung on the right side, but not on the left; the lung of the left side presenting nothing except a considerable degree of sanguineous engorgement. The pleuræ on both sides were quite uninjured, and there was no fracture of the ribs. The chief alterations consequent on the injury were emphysema of the neck and trunk, effusion of air into the right cavity of the pleura, causing very remarkable compression of the lung, laceration of the right lung, with extravasation of blood about the roots of the great vessels, and congestion of the left lung. The most singular circumstance connected with

* Dublin Journal, September, 1840.

the case was the occurrence of such extensive laceration of the lung without rupture of the pleura, or fracture of the ribs. These parts were examined with great care, but no solution of continuity could be discovered. The effused air had first passed into the mediastinum, and subsequently into the cellular tissue of the neck and trunk. This was the fourth case of rupture of the lung, without fracture of the rib, which Mr. Smith had witnessed. The first case was that of a woman who had met with a severe accident, and in whom this condition was discovered after death. The next case was that of a dog which was killed near the Richmond Hospital, by a car passing over its body. Mr. Smith examined the body shortly after the accident, and found that, although there was not a single rib fractured, the pericardium was torn in various directions, and the lung extensively lacerated. The last case was that of a man brought into Jervis-street Hospital, who had general emphysema and rupture of the lung, without any injury of the ribs or pleura.

3. *Softening of both Lobes of the Cerebellum—Symptoms.*—Our readers are aware of the uncertainty that obtains in reference to the functions of the cerebellum. It is thought to be a regulator of motion, and to be connected with the venereal passion. The following facts do not seem to lend much confirmation to either notion.

The subject of the case was a young woman, æt. 26, unmarried, and who enjoyed perfect health until three months before her death, when she was attacked with intense headache, not referred to any particular part of the head, and accompanied by sickness of the stomach in the morning. When she applied at St. Vincent's Hospital, she was almost completely amaurotic, and had double vision, which continued until her death: her headache was intense, but intermitting: she suffered greatly from globus hystericus. Previous to her admission into the hospital, she had undergone a course of mercury; and while in hospital, she was again salivated with temporary benefit: under the use of mercury her pulse fell from about 90 to 72; and for some time her febrile symptoms underwent a marked improvement. She had no paralysis during the whole course of the disease; the prominent symptoms were intense, but not constant headache, globus hystericus, double vision, amaurosis, and strabismus. She died rather suddenly about three months after the first appearance of her symptoms. Upon examination after death, the anatomical characters of chronic inflammation of the membranes of the brain were found, *and both lobes of the cerebellum were softened* nearly throughout their whole extent, and of a pale rose colour.

4. *Flaccid state of the Heart in Fever.*—Dr. Stokes has observed that, in certain cases of typhus, the sounds of the heart are greatly diminished, so that one or both become more or less indistinct. In such cases, he has stated that there is a specific change, or softening of the heart's substance. Dr. Graves exhibited the heart of a man labouring under typhus without maculæ. He was admitted into the Meath Hospital on the fourteenth day of his illness. It would be unnecessary to give any detail of the symptoms, further than to state, that on admission his pulse was strong and dicrotous, continued so for some days, and did not lose this character until five or six days before death. He died on the thirty-third day of fever. During the course of the disease, the chief symptoms were a dry tongue, dicrotous pulse, general bronchitis, with congestion of the lung, and diarrhœa. Some thought that one of the sounds of the heart was inaudible; but this was dubious. The man died on the thirty-third day of the fever. There was a general flaccidity of the heart, but no evident specific softening of its substance.

5. *Co-existence of Scirrhus of the Pylorus and Tubercles of the Lungs.*—Mr.

Smith presented the recent parts in this case, one of the lungs contained a tubercular cavity, which had evidently existed for a considerable time, and several scattered groups of tubercles were found in both lungs. Along the lesser curvature of the stomach, and completely encircling the pylorus, there was a large cancerous ulcer, the surface and circumference of which presented numerous fungous growths, the mesenteric glands were much enlarged, and filled with a white, cheesy matter, of a scrofulous character.

Similar cases have been mentioned by Bayle.

6. *Is Bright's Kidney the cause of Albuminous Urine?*—Dr. Graves exhibited a case of granular kidney, and detailed the particulars of the case. The Reporter states:—A question then arose, whether that state of the kidney termed "Bright's kidney" was the cause of albuminous urine, or whether it was to be looked upon in an opposite point of view, and regarded as the consequence. Dr. Graves said he was inclined to adopt the latter opinion for various reasons. He had seen so many persons cured of albuminous urine under various circumstances, that he should hesitate in ascribing this condition to a permanently disorganized state of the kidneys. A remarkable instance of this occurred in the person of Staff Surgeon Finnie, Surgeon to the Military School at the Park; about six months ago he was attacked with pleuro-pneumonia of the left lung, of a very severe character, and speedily followed by anasarca. About the time the anasarca was making its appearance, his urine became highly albuminous, and continued so for nearly six weeks. About the time when the pectoral symptoms were about to yield to treatment, the anasarca and ascites began to disappear, and at the same time the urine began to lose its albuminous character. In this case the pectoral symptoms were so severe, that for some time Dr. Graves indulged but faint hopes of his recovery. The antiphlogistic treatment pushed to its furthest extent, mercury to salivation, and repeated and powerful vesication succeeded in removing the disease, and with it all trace of albumen in the urine. The case occurred about six months ago, and the gentleman has remained quite well ever since, and without manifesting any symptoms whatever of a return to the albuminous diathesis.

It should be borne in mind, that in the dropsical diathesis there is a tendency to the secretion of water loaded with albumen, not only in the kidney, but also into the cellular membrane and serous cavities. It is rather difficult to conceive, that when the general system takes on this diseased action, the kidneys should be exempt, or that the kidneys should become affected with organic disease, in order to pour out albumen, while other tissues and organs can assume the same function without any structural alteration. Why should the kidneys alone become changed, while other parts retain their organic constitution? Dr. Graves said that he thought a few observations on this point would not be out of place. The Profession were in general aware, that modern discoveries have shown that the cortical portion of the kidney consists of an immense number of very minute tubes convoluted on each other, blending, and lying in apposition with the ultimate ramifications of the the arterial capillaries, through the parietes of which the urine is separated from the blood, making its first appearance in the minute tubes of the cortical substance. Now what are the chief constituents of urine? water, salts, and various acids, as the phosphoric, nitric, &c. Now if the nascent principles of the urine are secreted into these minute tubes in company with nascent albumen, the latter will be inevitably coagulated by some of the above-mentioned acids, and if this process be often and continually repeated, it is not surely a very improbable result, that these tubes may ultimately become filled with coagulated albumen, a fact observed by Valentin.

We quite agree with Dr. Graves that albumen in the urine is not necessarily indicative of serious disorganization of the kidneys. Every physician and surgeon must see cases of albuminous urine cured. But, whilst we allow this we must say that, in the great majority of instances, the symptom must be deemed a serious one, and, probably, the sign of that state of kidney which will end in organic alteration—a state akin to an inflammatory one.

7. *State of the Lungs in Hooping-cough.*—A child died of hooping-cough, following bronchitis. The right lung was chiefly affected. It had occupied more than its natural share of the cavity of the thorax, and had pushed the opposite lung towards the left side. It exhibited subpleural vesicles, and other evidences of emphysema; but the most remarkable circumstance connected with it was the existence of pneumonic inflammation affecting not the central portion of the lung, but merely the margins in which the induration of the pulmonary tissue was obvious.

8. *True Aneurysm of the common Iliac Artery, opening into the common Iliac Vein.*—Mr. Adams exhibited a specimen which bore some resemblance to the case recorded by Mr. Symes. It was a fusiform dilatation of the common iliac artery, with a communication between it and the common iliac vein. It appeared to be of several years standing, and to have caused the patient a good deal of distress. Some time ago he fainted while walking, and was carried home in a state of syncope, and died shortly afterwards. He had for a long time before death noticed a tumor, attended with a purring sensation in the lower part of the abdomen on the right side. On examination after death, Mr. Adams discovered a fusiform dilatation of the common iliac artery, and on one side of it there was a small opening about the size of a goose-quill, by which it communicated with the corresponding vein. Mr. Adams said he had examined the interior of the artery at the dilated portion, and could not find any solution of continuity in the lining membrane, except when the sac had opened into the iliac vein. He was therefore induced to look upon the preparation as being in the first instance a specimen of true fusiform aneurysm, and secondly, as affording an instance of spontaneous varicose aneurysm.

---

### MR. SMITH ON THE DIAGNOSIS AND PATHOLOGY OF FRACTURES OF THE NECK OF THE FEMUR.

One of the most remarkable memoirs we have lately read, is by Mr. Robert William Smith, lecturer on surgery at the Richmond Hospital School of Medicine, on Fractures of the Neck of the Femur. It is contained in the number of the Dublin Journal for September, 1840, and will amply repay the perusal of the surgeon. The memoir itself occupies seventy pages, and we must refer those who would become acquainted with the subject in detail, to it. We can do no more than present the conclusions which Mr. Smith draws from the facts. These consist in the post-mortem examination of fifty specimens of fracture of the neck of the femur, forty-two of which he relates. He deduces, then, the following conclusions :—

1. A slight degree of shortening, removable by the extension of the limb, indicates a fracture within the capsular ligament.

2. The degree of shortening, where the fracture is within the capsular ligament, varies from a quarter of an inch to one inch, or one inch and a half.

3. The degree of shortening, when the fracture is within the capsule, varies chiefly according to the extent of laceration of the fibro-synovial folds which invest the neck of the femur.

4. In some cases of intracapsular fracture the injury is not immediately followed by shortening of the limb.

5. This absence of shortening is generally owing to the integrity of the fibro-synovial folds.

6. In such cases the retraction of the limb may occur suddenly, many weeks after the receipt of the injury.

7. This sudden retraction of the limb, which indicates a fracture within the capsule, is, in general, to be ascribed to the accidental laceration of the fibro-synovial folds.

8. The degree of shortening, when the fracture is external to the capsule and not impacted, varies from one inch or one inch and a half to two inches or two inches and a half.

9. When a great degree of shortening occurs immediately after the receipt of the injury, we usually find a comminuted fracture external to the capsule.

10. The extracapsular fracture is generally accompanied by fracture with displacement of one or both trochanters.

11. The intracapsular *impacted* fracture is generally accompanied by fracture without displacement of one or both trochanters.

12. In such cases the fracture of the processes unites more readily than that of the cervix.

13. The degree of shortening, when the fracture is impacted, varies from a quarter of an inch to one inch and a half.

14. The exuberant growths of bone met with in these cases have been by many erroneously considered to be merely for the purpose of supporting the acetabulum and the neck of the femur.

15. The difficulty of ascertaining crepitus, and of restoring the limb to its natural length are the chief diagnostic signs of the impacted fracture.

16. The position of the foot is as much influenced by the obliquity of the fracture and the relative position of the fragments, as by the action of the muscles.

17. Inversion of the foot may occur in the intracapsular, extracapsular, or impacted fracture of the neck of the femur.

18. When in the intracapsular fracture the lower fragment is placed in front of the upper, the foot is usually inverted.

19. When in the extracapsular fracture with impaction, the superior is driven into the inferior fragment, so as to leave the greater portion of the latter in front of the former, the foot is generally inverted.

20. In cases of comminuted extracapsular fracture without impaction, but with separation and displacement of the trochanters, the foot may be turned either inwards or outwards, and will generally remain in whatever position it has been accidentally placed.

21. The consolidation by bone of the intracapsular fracture is most likely to occur, when the fracture is also impacted.

22. Severe contusion of the hip-joint, causing paralysis of the muscles which surround the articulation, is liable to be confounded with fracture of the neck of the femur.

23. The presence of chronic rheumatic arthritis may not only lead us to suppose that a fracture exists when the bone is entire, but also when there is no doubt as to the existence of fracture, may render diagnosis difficult as to the seat of the injury with respect to the capsule.

24. Severe contusion of the hip-joint, previously the seat of chronic rheumatic arthritis, and the impacted fracture of the neck of the femur, are the two cases most liable to be confounded with each other.

25. Each particular symptom of fracture of the neck of the femur, separately considered, must be looked upon as equivocal: the union of all can alone lead to correct diagnosis.

### MR. DONOVAN ON THE HYDROCYANOFERRATE OF QUININA.*

This salt has been brought forward by Signior Bertozzi, of Cremona, as a substitute for sulphate of quinina, where that fails. Doctors Zaccarelli and Carioli have confirmed his statements and anticipations. Mr. Donovan gives directions for procuring the salt, for which we must refer to our contemporary.

The hydrocyanoferrate of quinina, when in small fragments, is of a pea-green colour; its taste is intensely bitter; it dissolves in cold, but better in hot alcohol, and is precipitated almost entirely from the solution by water. In prescription, it would be an error to promote its solution in water by means of dilute sulphuric acid, as is done in the case of sulphate of quinina; the salt would be decomposed by this acid, and the solution would become blue. It ought not to be prescribed with tincture of cinchona, and consequently not with infusion or decoction. The dose given by Doctor Zaccarelli, was equal to three grains and a half troy, repeated according to necessity.

Although this febrifuge is precipitated by water from its alcoholic solutions, it separates in the state of so fine a powder, and remains so long suspended, that it will answer for exhibition very well in this state. The following formula will be found convenient:

℞ Hydrocyanoferratis quininæ grana quatuor,
Spiritûs rectificati drachmam. Solve.
Adde aquæ, vel
Misturæ camphoratæ drachmas septem. Misce fiat haustus, ut res nata sit, phialâ prius agitatâ, sumendus.

In pills

℞ Hydrocyanoferratis quininæ grana viginti quatuor,
Mucilaginis gummi Arabici q. s.
fiat massa quam divide in pilulas duodecim.

These pills will be of a proper size, and two of them will constitute a dose; to be repeated according to the discretion of the prescriber.

Mr. Donovan thinks the liquid form the better. He recommends, and so do we, the medicine to the profession.

---

### MR. DONOVAN ON COD-OIL.†

It appears that cod-oil has been a good deal used, of late, in France and Germany, in certain scrofulous cases. They *say* that when properly administered cod-oil cures scrofula of the bones, marasmus, and chronic arthritis of a scrofulous or rheumatic form. Caries, accompanied by a sore and swelling of the soft parts, requires the treatment with oil to be seconded by local applications, such as compression, and ioduretted alcoholic fomentations, cod-oil is of no avail against gouty arthritis, or swelling of any lymphatic glands but those of the abdominal cavity; its action seems doubtful or null in scrofulous phthisis when at all advanced. To produce advantageous results, in any disease, the use of cod-oil must be persevered in for several months, in doses of three or four table-spoonfuls daily.

Now, if this be all true, cod-oil is no bad thing, and it would be well to have it as good as can be got. Perhaps it should *not* taste exactly like train-oil, as that might make one sick, if it did nothing else. So Mr. Donovan has perfected the process for its preparation, and made cod-oil a very respectable oil to take.

---

* Dublin Journal, July 1, 1840.

† Ibid.

Take, says he, any quantity of livers of cod, throw them into a very clean iron pot, and place it on a slow fire, stir them continually until they break down into a kind of pulp: water and oil will have separated. When a thermometer plunged in the pulp will have risen to 192°, the pot should be taken from the fire, its contents transferred to a canvass bag, and a vessel placed underneath. Oil and some water will run through. After twenty-four hours, separate the former by decantation, and filter it through paper.

This oil, thus prepared, is of a pale yellow colour; its smell is weak, and resembles that of a cod boiled for the table when in excellent condition. Its taste is bland, by no means disagreeable, and as might be expected, is totally free from rancidity. It is very liquid. Its specific gravity in my trials was 0.934, although in all the published tables of specific gravities it is stated to be 0.923. In cold weather it deposits much stearine, and this ought not to be separated.

The product of pure oil is very variable. He has obtained so much as a gallon (wine measure) from twenty-eight pounds of livers, the produce of fifty cods. Sometimes the livers will afford much less. The runnings of the first heat only should be used: a second heat will supply more oil, but it will be comparatively strong-smelling, ill-tasted, and deep-coloured. The above estimate is true only when the fish is in the best season, and fully grown. Towards the close of the season the produce will be less. The livers of some cods are flaccid and lie flat without plumpness on a plane surface. These afford a deficient quantity of oil, a brown, strong-smelling quality, and a large portion of brown water: they are totally unfit for use, and their oil is disgusting. The livers are often found diseased and dark-coloured; such afford a very bad oil, and are of course to be rejected.

On the west coast of Ireland, they consider the beginning of the year the best season, and on the east the month of November for the cod-livers. Thus, concludes Mr. Donovan, in preparing cod-oil fit for medical purposes, three chief things are to be attended to: the livers must be perfectly healthy; they must be as fresh as possible, the least putrescency being injurious; and the heat at which the extrusion of the oil is effected must not exceed 192°.

---

### Rigidity of the Lower Jaw, cured by Division of the Anterior Portion of the Masseter Muscle.*

Dr. Mutter relates this case in our transatlantic contemporary. In January, 1839, he was requested to visit Harriett Wolcott, aged 16, for an affection of the mouth under which she had been labouring since her fourth year, and which followed inflammation of the cheek. Upon examination the left side of the jaw appeared much smaller than the right, and the integuments about the chin (on the same side) were much more closely connected with the bone, than is usual. The whole left half of the inferior maxillary bone, is in reality smaller than the right, and instead of presenting the natural angle at the junction of the ramus with the horizontal portion, is rounded off, so that it appears much straighter than it should be. There is no scar, nor any evidence of previous ulceration either within or without the cheek, neither does there exist any adhesion between the cheek and bone, but a strong fibrous band formed of the anterior portion of the masseter muscle was readily detected by introducing the finger between the teeth and cheek. This band is so short as effectually to prevent any thing like motion (except to a very limited extent) of the inferior maxillary bone, and the space between the upper and lower teeth is so small that an instrument

* American Journal of Medical Sciences, May 1840.

only three lines in thickness can freely be introduced between them. The patient is of course unable to chew, and has lived almost exclusively upon broths; nor can she protrude the tongue; and her teeth, in consequence of the impossibility of passing a brush between them, are in a very bad condition. Her articulation is also exceedingly defective.

Dr. Mutter thought it adviseable to divide the masseter muscle, and afterwards, by a lever, gradually force the jaws apart.

"Having placed my patient in a good light, I passed the forefinger of my left hand along the space between the cheek and teeth until it was arrested by the band already alluded to. She was then requested to open her mouth as much as possible in order that the band might be put upon the stretch.—Using my finger as a director, I next passed along it, an instrument shaped like a common gum lancet, though larger, and having but one cutting edge, until its point rested behind the band. By pressing firmly upon the handle, the blade was made to penetrate the masseter muscle about its lower third, until the point could be felt between the muscle and integument. The band was then divided by drawing the knife forwards and at the same time directing it outwards and downwards. The section was indicated by a slight snap, and the propriety of the operation at once made manifest by the improvement in the case. For example the finger could now be passed between the teeth, which before the operation was impossible."

Dr. Mutter now introduced the lever that he had prepared with a screw, and afterwards gradually separated the jaws by means of it. At the end of six weeks, when the report closes, the space between the teeth was one inch and three lines, and the patient could *chew* as well as ever.

---

### CASE OF IMPERFORATE VAGINA, AND MENSTRUATION (FROM THE BLADDER?)*

Ellen S——n, æt. 40, and married ten years, presented herself as an out-patient at St. Thomas's Hospital, under the care of Dr. Cape, on Friday, April 24, 1840. She complained of a hard and painful tumor, about the size of a melon, situated in the uterine region, which could be felt above the pubis. She had no discharge, and her general health was not impaired. On examination per vaginam being attempted, no vagina could be found. She was then requested to retire to one of the female wards, where she submitted to an ocular examination; the labia, nymphæ, clitoris, and all the external organs of generation, were quite normal, with the exception of the vagina, which was totally imperforate, not amounting even to a cul-de-sac; being perfectly flat and unyielding, with a granulated red surface. On examining per anum, the tumor could be very distinctly felt, painful on pressure, and apparently of a subcartilaginous character. She has menstruated regularly ever since she was thirteen, the period lasting four days, and of the usual quantity.

On the 15th of May, the catamenia being present, it was thought a good opportunity for ascertaining whence the discharge flowed, as, on the previous examination, the only aperture visible was the orifice of the urethra; when it was seen distinctly exuding guttatim from the meatus urinarius.

---

* Med. Gazette, June 26, 1840.

### Malformation of the Œsophagus.*

This case is communicated to our contemporary by Mr. Mellor, of Charlton-upon-Medlock, near Manchester.

" In the month of August 1839, Mrs. P., the mother of four healthy children, was delivered of her fifth, a fine, well-formed infant, after a perfectly natural labour of a few hours' duration. When in due time the infant was put to the breast, it was observed that the nipple was scarcely retained in the mouth beyond a minute, when the little creature became apparently convulsive, and almost instantly rejected the nutriment which it had taken. On my subsequent visit I was made acquainted with the above particulars, when I felt disposed to assent to the opinion expressed by the mother, that flatulency might be the cause of the symptoms, and accordingly prescribed a simple carminative. This was almost immediately rejected, as the milk had been, the infant notwithstanding manifesting the greatest eagerness to supply its instinctive wants. Inferring the existence of some obstruction in, or malformation of the œsophagus, I attempted to pass a common bougie, which however proceeded only for a very short distance, and then became curved upon itself, apparently not having arrived further than the commencement of the œsophagus. Matters went on in this way for six days, no other evacuation having taken place from the bowels than the meconium, and the infant continuing to apply its mouth to the breast with no other result than that of a slight convulsive paroxysm so soon as the pharynx became filled, and the immediate repulsion of its contents. Early on the morning of the seventh day it died, when a post-mortem examination disclosed the existence of little more than the membranous pouch of the pharynx, which terminated in a cul-de-sac a little below the cricoid cartilage, no trace of the œsophagus being visible beyond this part. The stomach presented no deviation from its ordinary form and dimensions, with the exception of its cardiac orifice, where there existed a slight bulging at the part corresponding with the termination of the gullet, and which was firmly united to the diaphragm. Further, a probe, introduced at this aperture, could not be made to pass into the stomach. Between this point and the sternum not the slightest trace of œsophagus, or any bond of connexions whatever with the pharyngeal portion, existed. The stomach contained nothing but air and a little mucus; the other viscera appeared perfectly normal.

In juxta-position with the foregoing case of defective conformation, I may mention an instance lately communicated to me by Mr. Heath, a highly respectable practitioner of this town. In this, the infant, which was of ordinary size, presented symptoms very analogous to those above described, and survived until the eighth day. On inspection after death, the duodenum was found obliterated to the extent of an inch or more, no further malformation having been observed."

---

### Analyses of the Mineral Waters at Brighton.

We extract the following Table from a pamphlet recently published by Mr. Pickford, on the Artificial Mineral Waters prepared at Brighton. So many invalids are sent to that watering place, that it may be useful to many of our readers to see, at a glance, the composition of the waters prepared there.

---

* Med. Gazette, June 26, 1840.

TABLE OF ANALYSES OF SOME OF THE PRINCIPAL MINERAL WATERS PREPARED AT THE ROYAL GERMAN SPA, BRIGHTON.

| Grs. of Anhydrous Ingredients in one pound Troy. | Carlsbad. | Ems. | Marienbad | Kissigen. | Saratoga. | Schlesischer. | Eger. | Pyrmont. | Spa. | Pullna. | Seidschutz |
|---|---|---|---|---|---|---|---|---|---|---|---|
| Carbonate of Soda....... | 7·2712 | 8·0625 | 5·3499 | .. | 0·8261 | 7·6211 | 0·8914 | .. | 0·5531 | .. | .. |
| Ditto of Lithia .......... | 0·0150 | 0·0405 | 0·0858 | .. | .. | .. | 0·0282 | .. | .. | .. | .. |
| Ditto of Baryta.......... | .. | 0·0022 | .. | .. | .. | .. | .. | .. | .. | .. | .. |
| Ditto of Strontia......... | 0·0055 | 0·0080 | 0·0028 | 0·0592 | 0·0672 | 0·0170 | 0·0023 | .. | .. | .. | .. |
| Ditto of Lime ........... | 1·7775 | 0·8555 | 2·9509 | 4·8180 | 5·8531 | 1·5464 | 1·3501 | 4·7781 | 0·7387 | 0·5775 | 5·1045 |
| Ditto of Magnesia........ | 1·0275 | 0·5915 | 2·0390 | 1·3185 | 4·1155 | 1·5496 | 0·5040 | .. | 0·8421 | 4·8045 | 0·8235 |
| Proto-Carb. of Manganese. | 0·0048 | 0·0028 | 0·0288 | 0·0121 | 0·0202 | 0·0026 | 0·0322 | 0·0364 | 0·0389 | .. | 0·0032 |
| Proto-Carb. of Iron ...... | 0·0208 | 0·0120 | 0·1319 | 0·1397 | 0·0173 | 0·0356 | 0·1762 | 0·3213 | 0·2813 | .. | 0·0095 |
| Sub-Phos. of Lime ....... | 0·0012 | .. | .. | .. | .. | .. | 0·0172 | .. | 0·0102 | 0·0026 | 0·0117 |
| Ditto of Alumina ........ | 0·0019 | 0·0014 | .. | .. | .. | .. | 0·0092 | 0·0110 | 0·0064 | .. | 0·0088 |
| Sulphate of Potassa ...... | .. | 0·4050 | .. | 1·2540 | 0·1379 | 0·3160 | .. | 0·0314 | 0·0593 | 3·6000 | 3·6705 |
| Ditto of Soda............ | 14·9019 | .. | 28·5868 | .. | .. | 2·5106 | 18·3785 | 1·6092 | 0·0281 | 92·8500 | 17·6220 |
| Ditto of Lithia .......... | .. | .. | .. | .. | .. | .. | .. | 0·0067 | .. | .. | .. |
| Ditto of Lime........... | .. | .. | .. | 5·5485 | .. | .. | .. | 5·0265 | .. | 1·9500 | 1·1287 |
| Ditto of Strontia......... | .. | .. | .. | .. | .. | .. | .. | 0·0154 | .. | .. | 0·0347 |
| Ditto of Magnesia........ | .. | .. | .. | .. | .. | .. | .. | 2·3684 | .. | 69·8145 | 62·3535 |
| Nitr: of Magnesia........ | .. | .. | .. | .. | 0·1004 | .. | .. | .. | .. | .. | 5·9302 |
| Chlor: of Ammonium .... | .. | .. | .. | 0·0364 | 0·0326 | 0·0164 | .. | .. | .. | .. | .. |
| Ditto of Potassium....... | .. | 0·0338 | .. | .. | 1·6256 | .. | .. | .. | .. | .. | .. |
| Ditto of Sodium ......... | 5·9820 | 5·7255 | 10·1727 | 39·3733 | 19·6653 | 0·8682 | 6·9229 | .. | 0·3371 | .. | .. |
| Ditto of Magnesium ... .. | .. | .. | .. | 3·6599 | .. | .. | .. | 0·8450 | .. | 14·7495 | 1·2225 |
| Bromide of Sodium....... | .. | .. | .. | 0·3331 | 0·1613 | 0·0051 | .. | .. | .. | .. | .. |
| Iodide of Sodium . ...... | .. | .. | .. | .. | 0·0046 | .. | .. | .. | .. | .. | .. |
| Fluoride of Calcium...... | 0·0184 | 0·0014 | .. | .. | .. | .. | .. | .. | .. | .. | .. |
| Alumina. .............. | .. | .. | 0·0023 | .. | 0·0069 | .. | .. | .. | .. | .. | .. |
| Silica .................. | 0·4329 | 0·3104 | 0·2908 | 0·1609 | 0·1112 | 0·2423 | 0·3548 | 0·3727 | 0·3739 | 0·1320 | 0·0900 |
| Total ................. | 31·4606 | 16·0525 | 49·6417 | 56·7136 | 32·7452 | 14·7309 | 31·6670 | 15·4221 | 3·2691 | 188·4806 | 98·0133 |
| Carbonic Acid Gas in 100 cubic inches. .. } .. | 58 | 51 | 105 | 96 | 114 | 98 | 154 | 160 | 136 | 7 | 20 |
| Temperature (Fahr.) | Sprud. 165°<br>Neu. 138°<br>Muhl. 128°<br>Ther. 122° | Kessel. 117°<br>Kranchen 84° | Kreutz. 53° | Ragozi. 53° | Congress. 58° | Obersalz. 58° | Franzens. 54° | 56° | Pouhon. 50° | 5 ° | 58° |
| Analysed by................ | Berzelius. | [illegible]truve. | Berzelius. | Struve. | Schweitzer | Struve. | Berzelius. | Struve. | Struve. | Struve. | Struve. |

### Influence of the Left Bronchus in closing the Ductus Arteriosus.*

Mr. T. W. King, Curator of the Museum of Guy's Hospital, advances the following hypothesis to account for the rapid closure of the ductus arteriosus after birth.

"In fœtal life the air-tubes contain only a little fluid; they are probably but partially expanded, while the circulation is nearly single and equable, the auricles pretty equally full at all times, and the ventricles equally powerful. The chest continues of limited capacity; the diaphragm high, and the lungs confined about the heart. We may now reflect upon the principal effects of the first inspiration: a general expansion is made, which is never again to have its commensurate collapse; and, although that fulness of the lungs (as to air) which may be regarded as necessary and permanent, be not obtained till later, it seems pretty evident that the first inspirations must have a far larger share than any others in effecting this condition. Now the trunk becomes straighter; the neck is no longer bent forwards on the breast, and the trachea is elongated; the diaphragm, and with it the heart, is considerably drawn down, while the lungs are generally expanded, but most particularly outwards: and one particular result of all these changes, I imagine to be, that the left bronchus is rendered more full and tense, and also raised at the same time that the ductus arteriosus is drawn down with some force, and perhaps with some disposition to elongation. In fine, it is to the sudden and intimate cross contact of these two tubes that I would mainly attribute the closing of the blood-vessel, which is in a manner bent over the bronchus, and has also a more oblique direction given to its communication with the aorta."

We confess that we are inclined to attach more importance to the indraught of blood into the lungs, which inspiration must occasion, than to the mere stretching of the bronchus. Our readers, however, will judge for themselves. Mr. King's view is certainly ingenious.

---

### Rupture of the Heart into the Pericardial Sac. Life for ten hours.

Dr. Stroud relates the following case.

*Case.*—Frederick P., aged 29, after anxiety and vexation endured for a considerable time, and after having been liable to profuse bleeding of the nose, during the spring season, for many years, was, on the non-occurrence of this bleeding, subject for six weeks to a sense of fulness in the head, with lassitude and somnolency. On the morning of April 27, 1839, after having returned from Covent Garden Market, he was seized with faintness, giddiness and vomiting, and insensibility. The pulse became imperceptible, and he was apparently in a dying state. He was promptly bled by Mr. Symes, to three pints, recovered a little, continued complaining of great tightness of the chest, and weight at the heart, but died the same evening.

The pericardium contained a quart of blood which had escaped through an opening in the right auricle just below the insertion of the vena cava superior, the edges of which were not attenuated or apparently ulcerated. The author accordingly suggests the prudence of relieving plethoric oppression, even where signs of structural disease in the sanguiferous system are not evident. In this patient the heart was large, and loaded with fat.—*Med. Gaz. June* 19, 1840.

---

* Med. Gaz. July 10, 1840.

### RESOLUSIONS OF THE MEDICAL ASSOCIATION OF IRELAND.

The following are the principal Resolutions of this young, but, perhaps, powerful body.

Dr. G. W. O'Brien, of Ennis, moved the fifth resolution, viz. :—Resolved—That the objects of the Association are—

1. To form a society for the protection of Medical Practitioners in all their just and legal rights:

2. To seek for a Legislative enactment, giving a permanent constitution to the Profession, and directing a competent and uniform standard of Education, and an equality of privileges for all persons who shall, in future, be permitted to practise medicine throughout the Empire, and—

3. To secure for the public, in future, the services of a scientific Apothecary, who shall be protected in the exercise of his Profession, and not engage in the practice of Medicine.

Dr. Purcell, of Carrick-on-Suir, moved the sixth resolution, viz. :—Resolved—That the Council be directed to prepare a Petition to Parliament, for the enactment of a measure which shall provide for the regulation and control of the Medical Charities; also praying that adequate funds shall be provided for their support.

Dr. Jagoe, of Kinsale, moved the seventh resolution, viz. :—Resolved—That the Council be directed to prepare Petitions to Parliament for suitable remuneration to medical men, when called upon to perform public services in courts of justice.

Dr. Cranfield, of Enniscorthy, moved the eighth resolution, viz. :—Resolved—That the Council be instructed to prepare Petitions to Parliament, praying for attention to the neglected subject of Medical Police, and for encouragement to medical men disposed to engage in the investigation of all matters concerning the public health; and that copies of these different petitions shall be forwarded to each Local Secretary, in order to procure signatures in all parts of Ireland.

Dr. Cane, of Kilkenny, moved the ninth resolution, viz. :—Resolved—That the following plan of General Medical Reform shall be supported by this association :—

The establishment, by law, of one Faculty, having three branches, one in each of the capitals of the Empire; such Faculty to include all Practitioners in Medicine, both Physicians and Surgeons: each Branch to be governed by a Representative Council, elected periodically by and out of, the whole body of the Faculty in each Kingdom. The Councils to have the power of making regulations for the government of the Profession, and also of admitting Members: no person being permitted to practise without being examined and licensed as a Member of the Faculty. The regulations of the three Councils to be similar and uniform, general conferences being, from time to time, held in order to preserve uniformity. This 'One Faculty' plan contemplates the establishment of a class of scientific Apothecaries to be examined and licensed as such under the direction of the Councils; also, that no Practitioner "shall be permitted to sell drugs, or to compound medicines, unless prescribed by himself, or by others in consultation with him, and for his own patients, except in rural districts, and by special license." Mr. Donovan's proposal for establishing a College of Pharmacy, might, with some modifications, be made to coincide with this portion of the 'One Faculty' plan. Institutions for teaching not to be connected with the licensing body of the faculty.*

---

* Med. Gaz. June 19, 1840.

### Removal of Part of the Sphincter for Prolapsus Ani.

M. Robert, reflecting on the inadequacy of excision of the prolapsed mucous membrane, as a curative measure, conceived the idea of removing some of the sphincter. A bad case presented itself in June, 1839. A washerwoman, aged 33, in St. John's ward, at La Pitié. The woman, when pregnant for the third time, had a prolapsus recti which was but temporary, and only occasioned a supportable degree of discomfort. Her fourth pregnancy brought on a prolapsus of the uterus, a permanent and very considerable prolapsus of the rectum, and a relaxation of the parietes of the abdomen. M. Roux now excised a prominent portion of the mucous membrane of the rectum. This somewhat relieved her; but the prolapsus soon increased; the fæces came away involuntarily, and she had pains in the loins, and the upper part of the thighs. This combination of symptoms confined the patient to her bed, and in June 1839 she was admitted into the Pitié. At that time the sphincter was so relaxed that four fingers could be easily introduced. The patient having been prepared by a progressive diminution of diet, and the use of opium, so as to produce as long a constipation as possible, M. Robert proceeded to operate in the following manner :—He made an incision on each side of the anus, beginning several millimétres from the middle of the aperture, and directed backwards to the point of the coccyx. The flap of skin between the two incisions was removed, together with the portion of the sphincter which it covered; and half the length of the muscle was thus removed. The two sides of the wound were united by three stitches of the twisted suture.

The case went on well. In the month of August the patient walked, and passed her stools voluntarily; but a small *bourrelet* of the mucous membrane had already protruded, and though, twice touched with the actual cautery, was not destroyed. Yet the cure has been a lasting one.*

---

### Leech Gathering in Russia.

One of the branches of industry prosecuted here is singular enough; it is the gathering of leeches for the Hamburg dealers. When talking with a person connected with this trade, we thought of Wordsworth's friend, of leech-gathering fame; but the collectors of the Ukraine do their work in such a wholesale, unpoetic way, that Wordsworth would not soil his verses with them. Having exhausted all the lakes of Silesia, Bohemia, and other more frequented parts of Europe, the buyers are now rolling gradually and implacably eastward, carrying death and desolation among the leeches in their course; sweeping all before them, till now they have got as far as Pultavia, the pools and swamps of which are yielding them great captures. Here a thousand leeches are sold for four roubles (3s. 4d.); at Hamburg, before reaching which one half die, the same number is sold for 120 roubles (near £5); and in England the country apothecary pays £9 and £12. 10s. for the quantity which originally cost only 3s. 4d. But of every thousand at least seven hundred die before reaching England.—*Bremner's Excursions in the Interior of Russia.*†

---

### Advantages granted to Naval Medical Officers.

We are glad to perceive from Her Majesty's Order in Council, which has been

* Med. Gaz. Aug, 28, 1840. † Ibid.

recently issued, that a meritorious class of public servants, the naval medical officers are to be henceforth placed, in respect to rank, designation, pay, and retirement, on a scale more nearly assimilated to that assigned to officers of the army medical department. The Lords of the Admiralty have accordingly been pleased to appoint Sir D. J. H. Dickson, M.D. Inspector of Hospitals; and Robert Armstrong, M.D. Deputy Inspector, to be attached to Her Majesty's Royal Naval Hospital at Plymouth; while Dr. Richardson with the former, and Drs. Mortimer and Liddell with the latter rank, are respectively appointed to Haslar Hospital. But the greatest boon granted by the change of regulations, is the privilege justly given to the Naval Assistant Surgeon to reckon the whole period of his full-pay service in support of his future claims for increased pay and retirement.

